# Prevalence of Metabolic Syndrome in COPD Patients and Its Consequences

**DOI:** 10.1371/journal.pone.0098013

**Published:** 2014-06-20

**Authors:** Marie-Kathrin Breyer, Martijn A. Spruit, Corrine K. Hanson, Frits M. E. Franssen, Lowie E. G. W. Vanfleteren, Miriam T. J. Groenen, Piet L. B. Bruijnzeel, Emiel F. M. Wouters, Erica P. A. Rutten

**Affiliations:** 1 Department of Respiratory Medicine, Maastricht University Medical Center + (MUMC+), Maastricht, the Netherlands; 2 Program Development Center (CIRO), Center of expertise for chronic organ failure, Horn, the Netherlands; 3 Department of Respiratory and Critical Care Medicine and Ludwig Boltzmann Institute for COPD and Pulmonary Epidemiology, Otto Wagner Hospital, Vienna, Austria; 4 Division of Medical Nutrition Education, School of Allied Health Professions, University of Nebraska Medical Center, Ohmaha, Nebraska, United States of America; 5 Inflammation Neuroscience and Respiratory, AstraZeneca, Molndal, Sweden; Children's National Medical Center, Washington, United States of America

## Abstract

**Background:**

The prevalence of metabolic syndrome in COPD patients and its impact on patient related outcomes has been little studied. We evaluated the prevalence of metabolic syndrome and clinical and functional characteristics in patients with COPD and healthy subjects.

**Methods:**

228 COPD patients and 156 healthy subjects were included. Metabolic syndrome was defined using criteria of the IDF. In all patients spirometry, body composition, functional exercise performance, and mood and health status were assessed. Groups were stratified for BMI and gender.

**Results:**

Metabolic syndrome was present in 57% of the COPD patients and 40% of the healthy subjects. After stratification for BMI, presence of metabolic syndrome in patients with a BMI ≥25 kg/m^2^ was higher than in healthy peers. Patients with metabolic syndrome and a BMI <25 kg/m^2^ had higher BMI, fat free mass index and bone mineral density, and a lower 6MWD than the BMI matched patients without metabolic syndrome. Spirometry, maximal ergometry, mood and health status, and blood gases were not different between those groups. In COPD patients with metabolic syndrome self-reported co-morbidities and medication use were higher than in those without.

**Conclusion:**

Metabolic syndrome is more prevalent in overweight or obese COPD patients than in BMI matched healthy subjects. Metabolic syndrome did not additionally impact patients' functional outcomes, but did impact the prevalence of co-morbidities.

## Introduction

Chronic obstructive pulmonary disease (COPD) is characterized by persistent airflow limitation that is usually progressive. Additionally, exacerbations and co-existing morbidities contribute to the overall severity in the individual patient [1]. Indeed, cardiovascular co-morbidities are common in COPD [2] and are associated with an increased mortality risk [3], [4]. So far, the underlying mechanisms are only partially understood. In addition to smoking, other factors may contribute including advanced age, medications, systemic inflammation and metabolic disturbances.

Metabolic syndrome is a common metabolic disorder defined as a complex of interrelated cardiovascular risk factors [5]. Metabolic syndrome is age dependent [6] and has been related to several other health conditions [7] and an increased mortality risk [8]. In addition, metabolic syndrome has clinically relevant negative effects on subjects exercise capacity [9], as well as on health status [10], while protective effects are described on bone mineral density (BMD) [11].

To date, the prevalence of metabolic syndrome in COPD patients compared to healthy subjects has been studied scarcely [12]. Marquis and colleagues reported an increased prevalence in 38 COPD patients compared to 34 healthy subjects (47% vs. 21%, respectively) [13]. A comparable prevalence of metabolic syndrome in COPD was reported by Watz and colleagues [14], but no healthy control group was included in this study.

COPD patients with metabolic syndrome are physically less active and have increased levels of systemic inflammation compared to COPD patients without metabolic syndrome [14]. To date it remains largely unknown whether and to what extent other clinical outcomes, like spirometry, functional exercise performance, mood and health status, and the prevalence of cardiovascular co-morbidity might differ between COPD patients with and without metabolic syndrome. Therefore, we think it is of great interest to get more insight of the impact of metabolic syndrome on COPD patients' clinical outcomes in order to better characterise this subgroup of COPD patients.

In the present study we evaluated the prevalence of metabolic syndrome in COPD patients compared to healthy subjects. We hypothesize that the prevalence of metabolic syndrome is higher in the patients. In addition, clinical and functional characteristics of COPD patients with and without metabolic syndrome were studied.

## Methods

### Study population

Data were prospectively collected in 228 clinically stable COPD patients and 156 healthy subjects from 2007–2012. All patients and 55 healthy subjects were recruited as part of the CIROCO study and 101 control subjects were recruited from another observational prospective study. For both studies, inclusion criteria for the COPD patients were: diagnosis of COPD, GOLD I–IV [15] and no respiratory tract infection or exacerbation at least 4 weeks prior to the study. Exclusion criteria for both groups were any kind of oncologic pathology less than 5 years prior to the study.

### Ethics statement

Study 1 (CIROCO study): Ethical approval number: 10-3-067. Medical ethical committee of the Maastricht University Medical Centre. Study 2 ethical approval number: 10-3-033, Medical ethical committee of the Maastricht University Medical Centre, www.controlled-trials.com, ISRCTN86049077. All participants gave written informed consent.

### International Diabetes Federation (IDF) definition of metabolic syndrome [5]

Waist circumference (WC ≥94 cm in European men or ≥80 cm in European women)

*Plus two of the following:*

- glucose >100 mg/dL (5.6 mmol/L) or previously diagnosed type II diabetes;
- triglyceride ≥150 mg/dL (1.7 mmol/L) or specific treatment for this lipid abnormality;
- high density lipoprotein (HDL) <40 mg/dL (1.03 mmol/L) in men or <50 mg/dL (1.29 mmol/L) in women or specific treatment for this lipid abnormality;
- systolic blood pressure ≥130 mmHg and/or diastolic blood pressure ≥85 mmHg or treatment of previously diagnosed hypertension;

### Lung function and arterial blood gases

Lung function parameters were collected using standardized spirometry (Masterlab, Viasys, Germany). The degree of severity of airflow limitation was classified according to the GOLD guidelines [15]. Arterial oxygen tensions (PaO2) and arterial carbon dioxide tension (PaCO2) have been analyzed in the patients only.

### Anthropometric parameters

Fat free mass index (FFMI) and bone mineral density (BMD) have been assessed using a dual x-ray absorptiometry (DEXA) scan (Lunar Prodigy; GE Healthcare, Madison, WI, USA). FFMI was calculated as: FFM in kilogram divided by squared height in meters. Low FFM was defined as FFMI ≤15 (females) or ≤16 (males) kg/m^2^. BMD has been defined by a T-score (hip and lumbar spine) >−1, Osteopenia −2.5 and −1, and osteoporosis by a T-score ≤−2.5.

### Exercise capacity and mood status

In patients peak cycling load has been assessed by incremental ergometry cycling test and functional exercise capacity by six-minute walking distance test (6MWD). The St. George Respiratory Questionnaire (SGRQ) has been used to assess disease-specific health status [16]. Assessment of mood status has been done using the Hospital Anxiety and Depression Scale (HADS) [17].

### Additional parameters

The Modified Medical Research Council (MRC) dyspnoea scale, subjects' smoking status, number of pack years, the use of long-term oxygen therapy (LTOT), the Charlson Co-morbidity Index (CCI) [18] and current pharmacological treatment were recorded.

### Statistical analyses

Besides whole group comparison, both the patient and healthy subject groups were stratified for BMI (cut-off of 25 kg/m^2^) and for gender to evaluate the prevalence of the metabolic syndrome. Categorical variables are described as frequencies, while continuous variables were checked for normality and described as mean ± SD or median (inter-quartile range; for pack years). Evaluation of group differences (patients vs. healthy subjects, patients with vs. without metabolic syndrome) in means for continuous variables was done using the Unpaired Student's t-test, and for categorical variables the chi-square test. Logistic regression was performed to test whether COPD patients had a higher risk of having metabolic syndrome after correction for age, gender and BMI. All analyses were performed using the Statistical Package for the Social Sciences (SPSS) version 20 for Windows. A *p*-value of ≤0.05 was considered significant.

## Results

### Prevalence of metabolic syndrome and its components

**Figure 1:**
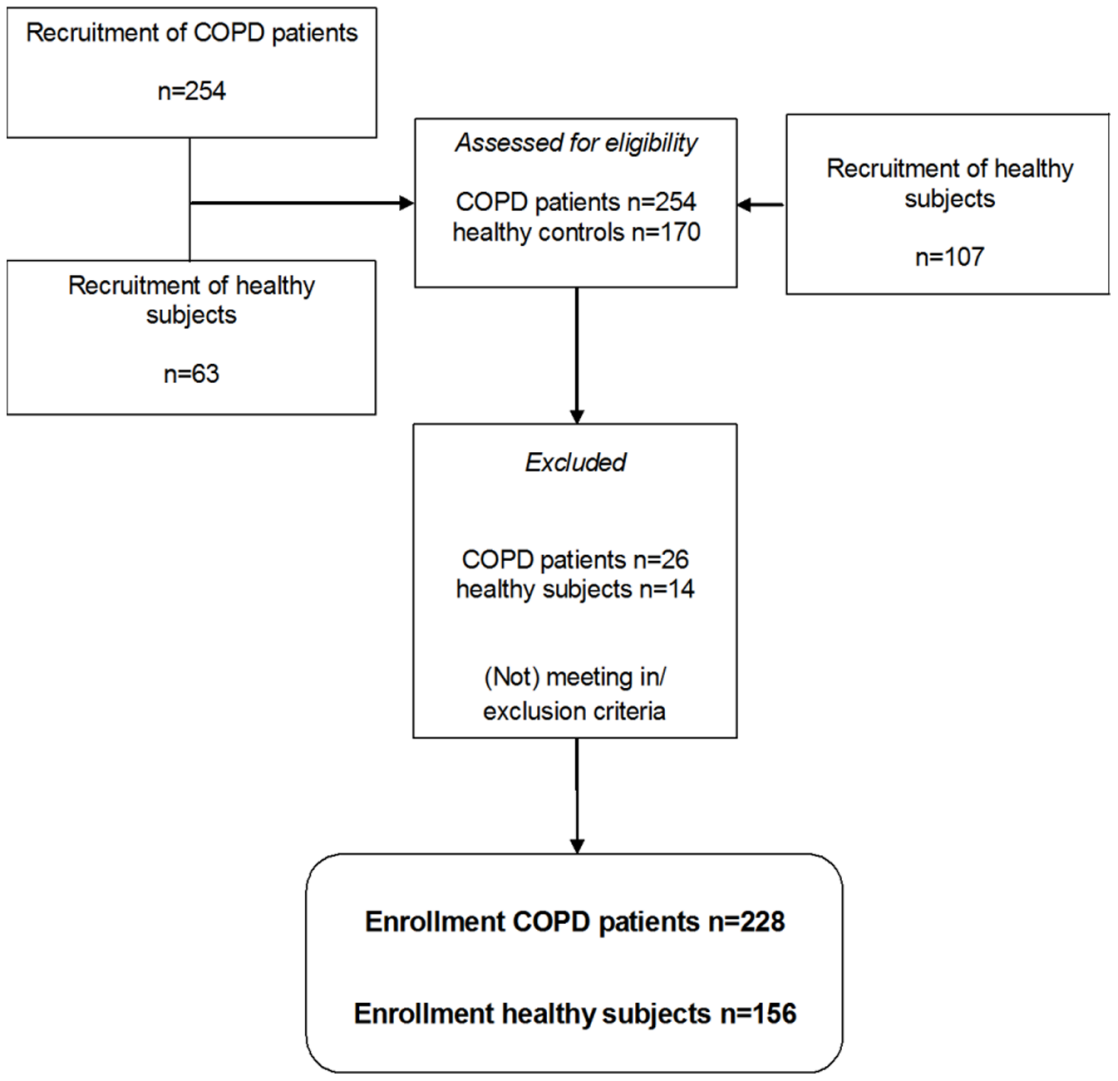
Patient flow diagram.

A diagram of participant flow is shown in **Figure 1**. The COPD patients were characterised by moderate to severe airflow limitation (**Table 1**). Compared to healthy, patients were slightly older, had a higher number of pack years and male gender was predominant. While BMI and FFMI were lower, WC was higher in the patients. Plasma HDL concentration and diastolic BP were lower in the patients. Prevalence of a high systolic and diastolic BP, one of the criteria of metabolic syndrome, was more prevalent in healthy than in the patients, but metabolic syndrome was more prevalent in the patients than in healthy subjects. After stratification for BMI: the subgroup of patients with a BMI ≥25 kg/m^2^ were matched for BMI with their healthy peers, but BMI was lower in the subgroup of patients with BMI <25 kg/m^2^ compared to their healthy peers (**Table 2**). Further, in the subgroups of subjects with BMI <25 kg/m^2^, WC was higher and plasma HDL concentration was lower in patients than in healthy, but the prevalence of the metabolic syndrome was comparable. In the logistic regression on metabolic syndrome, COPD patients had a 60% more chance of having metabolic syndrome than control subjects after correction for age, gender and BMI (**Table 3**).

**Table 1:** Baseline characteristics of the total study population.

	COPD patients (n = 228)	healthy subjects (n = 156)
Gender, %male	59	45*
age, years	63.7±7.1	60.1±7.4*
FEV_1_, %pred	52.8±18.6	120.4±15.8*
FVC, %pred	96.1±19.1	122.8±21.3*
FEV_1_/FVC	40.9±12.2	78.1±6.5*
smoking status		
never smoker, %	0	77*
former smoker, %	73	23*
current smoker, %	27	0*
Pack years, n#	43.0 (29.4–58.5)	3.5 (0.0–15.3)*
BMI, kg/m^2^	26.2±5.1	27.3±4.2†
FFMI, kg/m^2^	17.0±2.4	17.9±3.6*
Waist circumference, cm	98.5±14.3	93.9±10.7*
Plasma glucose level, mmol/L	5.8±1.2	5.6±0.7
Plasma triglyceride level, mmol/L	1.5±0.9	1.5±1.1
Plasma HDL level, mmol/L	1.7±0.5	1.8±0.5†
Systolic blood pressure, mmHg	138.6±21.4	142.7±20.3
Diastolic blood pressure, mmHg	82.2±9.7	84.3±9.1†
High waist circumference$, %	79	79
High glucose level$, %	49	45
High triglyceride level$, %	31	22
High HDL level$, %	10	8
High systolic blood pressure$, %	61	75*
High diastolic blood pressure$, %	33	46*
Metabolic syndrome$, %	57	40*

Data are presented: mean ± SD, percentages or ^#^median (interquartile range) if data were not normal distributed.

FEV_1_, forced expiratory volume in the first second; FVC, forced vital capacity; BMI, body mass index; FFMI, fat free mass index; HDL, high density lipoprotein.

$ according to the IDF.

Other symbols:

*p<0.01,

† p<0.05 compared with COPD patients.

**Table 2:** Body composition and criteria of metabolic syndrome of the study population by BMI.

	BMI <25 kg/m^2^		BMI ≥25 kg/m^2^	
	COPD patients (n = 97)	healthy subjects (n = 47)	COPD patients (n = 131)	healthy subjects (n = 109)
BMI, kg/m^2^	21.7±2.4	23.0±1.5*	29.4±3.9	29.1±3.5
FFMI, kg/m^2^	15.3±1.5	15.8±1.9†	18.3±2.1	18.8±3.7
Waist circumference, cm	87.4±10.6	83.3±7.7†	107±10.7	98.5±8.2*
Plasma glucose level, mmol/L	5.5±0.9	5.4±0.6	6.0±1.3	5.7±0.7†
Plasma triglyceride level, mmol/L	1.3±0.6	1.2±0.6	1.7±1.0	1.6±1.3
Plasma HDL level, mmol/L	1.8±0.5	2.0±0.5†	1.4±0.4	1.7±0.4
Systolic blood pressure, mmHg	136.5±19.9	135.1±18.5	140±22.3	146±20.2†
Diastolic blood pressure, mmHg	82.3±9.5	80.2±8.9	82.2±9.9	86.1±8.6*
High waist circumference$, %	49	57	100	88*
High glucose level$, %	41	36	55	49
High triglyceride level$, %	20	17	40	25†
High HDL level$, %	5	6	13	8
High systolic blood pressure$, %	55	55	66	83*
High diastolic blood pressure$, %	33	23	33	56†
Metabolic syndrome$, %	29	17	77	50*

Data are presented: mean ± SD or percentages. BMI, body mass index; FFMI, fat free mass index; HDL, high density lipoprotein.

$ according to the IDF.

Other symbols:

*p<0.01,

† p<0.05 compared with COPD patients.

**Table 3:** Logistic regression with metabolic syndrome as dependent variable.

	OR	CI	P-value
Age	1.03	0.99–1.06	0.11
Gender (male = 1)	0.58	0.37–0.92	0.02
BMI (BMI<25 kg/m^2^ = 1)	6.85	4.13–11.36	<0.01
Group (COPD = 1)	0.39	0.24–0.64	<0.01

The interaction term group*BMI was not significant, so it was excluded from the model. Abbreviations: OR: odds ratio; CI: 95^th^ percentile confident interval.

**Figure 2:**
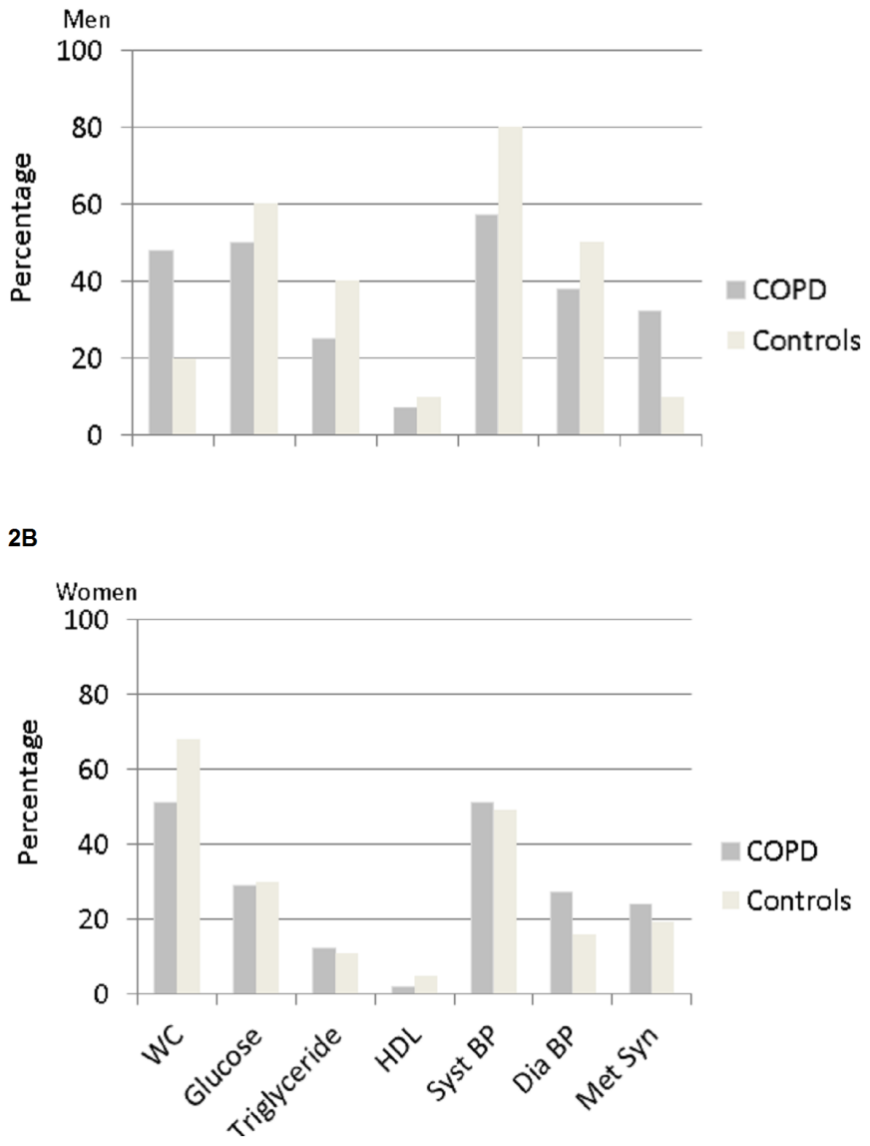
A and B. Prevalence of metabolic syndrome and its components in male and female COPD and healthy subjects with BMI <25 kg/m^2^. WC, waist circumference; HDL, high density lipoprotein; Syst BP, systolic blood pressure; Dia BP, diastolic blood pressure; Met Syn, metabolic syndrome.

**Figure 3:**
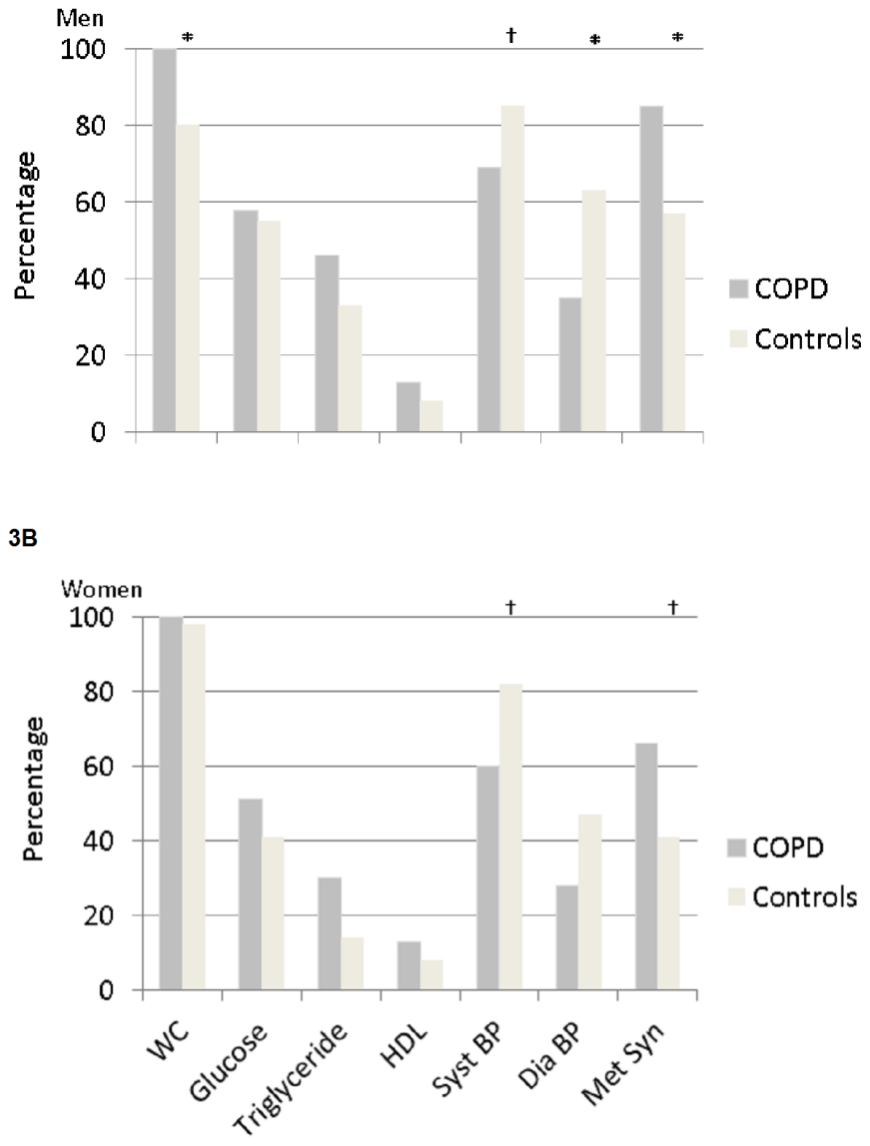
A and B. Prevalence of metabolic syndrome and its components in male and female COPD and healthy subjects by BMI >25 kg/m^2^. WC, waist circumference; HDL, high density lipoprotein; Syst BP, systolic blood pressure; Dia BP, diastolic blood pressure; Met Syn, metabolic syndrome. *p<0.01, †p<0.05 compared to COPD patients.

**Figure 2** **and** **3** provide an overview of the prevalence of metabolic syndrome and its components in patients and healthy subjects after stratification for gender and BMI. No differences were found between men and women with BMI <25 kg/m^2^ (**Figure 2, A and B**). In the subjects with BMI ≥25 kg/m^2^ (**Figure 3, A and B**), WC, systolic and diastolic BP, and the prevalence of the metabolic syndrome were different between men and women.

### Characteristics of patients with and without metabolic syndrome

Even after stratification for BMI, the markers of body composition, BMI and FFMI were higher in patients with compared to those without metabolic syndrome (**Table 4**). Further, in the patients with BMI <25 kg/m^2^, patients with metabolic syndrome had a lower prevalence of low muscle mass and a lower 6MWD compared to patients without metabolic syndrome. In addition, the proportion of patients using statins and BP lowering drugs was higher when having metabolic syndrome. In the patients with BMI ≥25 kg/m^2^, male gender was predominant and BMD of the lumbar spine was higher in the patients with compared to those without metabolic syndrome. Also the proportion of patients with co-morbidities, and the proportion of patients using statins, β-blockers and BP lowering drugs was higher in the patients with compared to those without metabolic syndrome. Other characteristics like spirometry, GOLD stages and combined COPD assessment, peak cycling load, prevalence of osteopenia/osteoporosis, use of LTOT, arterial blood gases, dyspnea, symptoms of anxiety and depression, and health status were similar between COPD patients with and without metabolic syndrome.

**Table 4:** Clinical and functional characteristics of the COPD patients by metabolic syndrome.

	BMI <25 kg/m^2^		BMI ≥25 kg/m^2^	
	Metabolic syndrome		Metabolic syndrome	
	with (n = 28)	without (n = 69)	with (n = 101)	without (n = 30)
age, years	64.3±6.6	62.9±7.2	64.1±7.2	63.7±7.3
male, %	64	55	65	40†
FEV_1_, % pred.	45.2±18.9	49.4±20.5	57.5±17.0	52.1±15.9
FVC, % pred.	92.8±23.3	97.5±17.7	95.8±19.4	97.1±16.9
FEV_1_/FVC, % pred.	35.6±9.9	37.8±13.4	44.6±11.6	40.2±9.3
GOLD I, II, III, IV, %	0, 43, 36, 21	7, 36, 41, 16	8, 59, 27, 6	7, 50, 40, 3
Combined COPD assessment A, B, C, D, %	32, 11, 4, 54	18, 23, 17, 42	23, 43, 2, 32	27, 30, 10, 33
BMI, kg/m^2^	23.2±1.3	21.1±2.5*	30.1±4.1	27.3±2.2*
FFMI, kg/m^2^	15.9±1.3	15.0±1.5*	18.6±2.2	17.1±1.5*
Low muscle mass, %	32	72*	4	7
BMD hip, g/cm^2^	0.80±0.10	0.80±0.15	0.89±0.13	0.89±0.23
BMD lumbar spine, g/cm^2^	1.04±0.16	1.01±0.20	1.14±0.18	1.05±0.16†
osteopenia , %	46	41	56	60
osteoporosis, %	50	49	19	20
6MWD, m	438.0±122.1	491.9±97.3†	460.4±95.5	474.6±125.3
6MWD, %pred	73.7±21.3	80.5±14.7	82.4±16.6	81.6±19.9
peak load, watts	68.0±19.4	68.2±23.8	81.7±28.6	78.2±38.4
peak load, %pred	56.6±31.5	55.1±23.8	61.9±25.8	65.9±26.9
smoking status				
never smoker, %	0	0	0	3
former smoker, %	64	64	79	73
current smoker, %	36	36	21	23
pack years, n#	38.5 (27.3–56.2)	43.8 (28.5–59.7)	43.8 (31.5–58.8)	38.3 (30.6–56.1)
LTOT, %	14	20	13	23
pH	7.44±0.03	7.43±0.03	7.43±0.02	7.44±0.09
PaCO_2_, kPa	5.3±0.7	5.3±0.6	5.3±0.6	5.5±0.6
PaO_2_, kPa	9.3±1.1	9.6±1.1	9.5±1.0	9.3±1.2
MRC	2.3±1.3	1.9±1.0	2.2±1.1	2.0±1.2
HADS, anxiety	6.0±3.8	6.5±4.3	6.0±3.8	7.2±4.2
depression	5.4±3.6	5.6±3.4	5.8±3.7	5.7±3.6
SGRQ, total score	52.2±16.2	49.8±18.1	52.7±16.9	50.3±19.7
symptom score	54.8±18.8	57.7±21.4	55.2±19.9	53.4±23.0
activity score	65.6±20.8	65.6±23.2	71.0±21.0	66.7±21.6
impact score	39.3±21.4	38.9±21.6	41.2±18.0	40.0±21.8
Charlson Co-morbidity Index				
CVD, %	32	22	39	27
Myocardial infarct, %	4	6	18	0†
Peripheral vascular disease, %	14	13	22	13†
Type II diabetes, %	7	3	8	3†
Medication use				
Inhaled CS, %	14	12	13	13
Oral CS, %	14	7	8	10
Statins, %	46	7*	47	3*
Beta-blockers, %	21	9	26	7†
Anti-diabetics, %	7	3	6	3
Insulin, %	0	0	3	3
Blood pressure lowering drugs, %	54	28†	61	17*

Data are mean ± SD or percentages or ^#^median (interquartile range) if data were not normal distributed.

FEV_1_, forced expiratory volume in the first second; FVC, forced vital capacity; BMI, body mass index; FFMI, fat free mass index; BMD, bone mineral density; 6MWD, six minute walking distance; LTOT, long term oxygen therapy; PaCO_2_, arterial carbon dioxide tension; PaO_2_, arterial oxygen tension; CVD, cardiovascular disease; inhaled and oral CS, inhaled and oral corticosteroids; MRC, modified Medical Research Council; HADS, Hospital Anxiety and Depression Scale; SGRQ, St. George Respiratory Questionnaire.

*p<0.01,

† *p*<0.05 compared to patients without the metabolic syndrome.

## Discussion

The present study reports on two important findings: first, metabolic syndrome is more prevalent in overweight to obese patients with COPD compared to BMI matched healthy subjects. In the present cohort, no difference in the frequency of metabolic syndrome was observed in low to normal weight patients and healthy subjects. Second, the presence of metabolic syndrome in COPD patients had no functional consequences, but it coincided with more cardiovascular co-morbidity and type II diabetes.

Fifty seven percent of the COPD patients were diagnosed with metabolic syndrome, a prevalence comparable with previous studies [13], [14]. However, this percentage was significantly higher compared to the healthy subjects, despite the presence of a lower BMI. Indeed, after stratification for BMI, the prevalence of metabolic syndrome was not significantly different in the subjects with BMI <25 kg/m^2^, but the prevalence of metabolic syndrome was significantly higher in the patients compared to the healthy subjects in the group with BMI ≥25 kg/m^2^. According to the IDF, the prevalence of metabolic syndrome is largely driven by abdominal obesity. In fact, it was recently shown that patients with obstructive lung disease have more visceral fat mass compared to healthy subjects [19]. Here, these findings are indirectly confirmed by the observation that even after stratification for BMI patients in both BMI sub-groups have higher WC than their matched healthy peers. It is uncertain why abdominal obesity is more prevalent in COPD patients compared to healthy subjects, but various factors including poor nutrition and an inactive lifestyle may play an important role [19]. It is remarkable that the prevalence of metabolic syndrome is only higher in the patients with BMI ≥25 kg/m^2^. Furthermore, cardiovascular co-morbidity was more prevalent in the patients with metabolic syndrome and BMI ≥25 kg/m^2^. These data imply that BMI contribute to the cardiovascular co-morbidity in COPD on top of metabolic syndrome.

The presence of metabolic syndrome was independent of the degree of airflow limitation. One other manuscript reported a slightly higher prevalence of metabolic syndrome in mild to moderate COPD patients [14]. In the present study most patients had COPD GOLD stage II or III, but no difference in the prevalence of metabolic syndrome within the various GOLD stages of COPD could be identified. Indeed, in the general population metabolic syndrome is associated with restrictive ventilatory patterns but not with obstructive lung function impairments [20]. This association is mainly driven by abdominal obesity, which is particularly present in patients with moderate airflow obstruction.

In patients with BMI <25 kg/m^2^, patients with metabolic syndrome had lower 6MWD compared to the patients without metabolic syndrome. This is a surprising finding as the prevalence of low muscle mass, which is associated with decreased exercise tolerance [21], was lower in the patients with metabolic syndrome. On the other hand, metabolic syndrome contributes to a lower exercise capacity assessed by treadmill test in healthy subjects [9], [22] and lower physical activity levels have been found in COPD patients with compared to those without metabolic syndrome [14]. However, differences in body composition were not taken into account as possible confounder. Interestingly, in the patients with BMI ≥25 kg/m^2^ metabolic syndrome did not affect functional exercise performance.

In the present study the prevalence of osteoporosis in COPD patients ranges from 20% up to 50% depending on BMI. To date no data are available on levels of BMD in COPD patients with metabolic syndrome. In the present study we did not find an association between the prevalence of metabolic syndrome and the prevalence of osteoporosis, but we did find a higher BMD of the lumbar spine in the patients with BMI ≥25 kg/m^2^ and metabolic syndrome. In the NHANES study, performed in the general population it was shown that subjects with metabolic syndrome have higher BMD compared to subjects without metabolic syndrome (after correcting for multiple confounders) and these results were mainly driven by an increased abdominal obesity [11]. Indeed, even a protective effect of criteria of metabolic syndrome against non-vertebral fractures has been described in patients with type II diabetes [23]. This effect could be partly explained by an increased mechanical load on the cortical skeleton [24] and increased insulin levels promoting bone formation due to an increased fat mass [25]. There aside, in COPD it is already shown that circulating leptin, a cytokine produced and secreted by adipocytes, appears to act as a mediator between fat mass and bone mass [26]. More studies should be performed to investigate whether metabolic syndrome in COPD could have a protective effect on osteoporosis.

COPD is known to have a negative impact on the disease-specific health status and the effect increases even more with increased disease severity [27]. Identifying factors related to a decreased health status in COPD are, besides lung function impairment [27], exercise intolerance [28], dyspnoea, anxiety [29], and body composition [30]. In general chronic conditions, such as type II diabetes, hypertension as well as dyslipidaemia are well known to affect patients' health status. Thus, it can be expected that subjects having metabolic syndrome also suffer from an impaired health status [31], [32]. In COPD co-morbid conditions might lead to a further decrease in health status. However, results from our study show that COPD patients having metabolic syndrome are not additionally affected in their disease-specific health status.

Finally the latest global strategy for the diagnosis, management and prevention of COPD (GOLD) highlighted the importance of co-morbidities in COPD. In particular, cardiovascular diseases, lung cancer, osteoporosis, depression, and metabolic disorders, such as type II diabetes, as they have a significant impact on prognosis and some of them even have been found to be the most frequent cause of death in mild COPD [1]. The here presented results indicate that having metabolic syndrome increases the co-morbidity index, particularly in those patients with an overweight or obese BMI. Therefore metabolic syndrome has to be identified and treated appropriately in patients with COPD.

The following methodological considerations have to be taken into account: firstly, healthy subjects were slightly younger than the COPD patients. However, it is unlikely that a mean age difference of about 3 years explains the difference in the prevalence of the metabolic syndrome. Secondly, blood pressure of the healthy was relatively high. Indeed, hypertension was the most mentioned co-morbidity among the healthy. Further, subjects were not allowed to take their blood lowering drugs before the tests. Thirdly, no metabolic effects of metabolic syndrome are taken into account in the present study. Nevertheless, there is evidence that metabolic syndrome is associated with disturbed adipokine metabolism, insulin resistance [33], and with increased systemic inflammation [14]. These studies did however not include healthy subjects and future research has to unravel whether this is a COPD specific effect. Fourth, the present study had a cross-sectional study design and longitudinal studies are warranted to investigate the long term effects of metabolic syndrome on cardiovascular and other morbidities in patients with COPD.

In conclusion, in contrast to normal weight patients, metabolic syndrome is more prevalent in overweight and obese patients with COPD compared to BMI matched healthy subjects. Both, lung function impairment and functional parameters did not differ between COPD patients with and those without metabolic syndrome. Additionally, the prevalence of osteoporosis and disease-specific health status were not altered by the presence of metabolic syndrome, while metabolic syndrome has shown to contribute to an increased co-morbidity index.

## References

[1] Barnes PJ, Celli BR (2009) Systemic manifestations and comorbidities of COPD. Eur Respir J 33: 1165–1185. 19407051 10.1183/09031936.00128008

[2] Curkendall SM, DeLuise C, Jones JK, Lanes S, Stang MR, et al (2006) Cardiovascular disease in patients with chronic obstructive pulmonary disease, Saskatchewan Canada cardiovascular disease in COPD patients. Ann Epidemiol 16: 63–70. 16039877 10.1016/j.annepidem.2005.04.008

[3] Mannino DM, Thorn D, Swensen A, Holguin F (2008) Prevalence and outcomes of diabetes, hypertension and cardiovascular disease in COPD. Eur Respir J 32: 962–969. 18579551 10.1183/09031936.00012408

[4] Divo M, Cote C, de Torres JP, Casanova C, Marin JM, et al (2012) Comorbidities and risk of mortality in patients with chronic obstructive pulmonary disease. Am J Respir Crit Care Med 186: 155–161. 22561964 10.1164/rccm.201201-0034OC

[5] Alberti KG, Zimmet P, Shaw J (2006) Metabolic syndrome–a new world-wide definition. A Consensus Statement from the International Diabetes Federation. Diabet Med 23: 469–480. 16681555 10.1111/j.1464-5491.2006.01858.x

[6] Eckel RH, Grundy SM, Zimmet PZ (2005) The metabolic syndrome. Lancet 365: 1415–1428. 15836891 10.1016/S0140-6736(05)66378-7

[7] Isomaa B, Almgren P, Tuomi T, Forsen B, Lahti K, et al (2001) Cardiovascular morbidity and mortality associated with the metabolic syndrome. Diabetes Care 24: 683–689. 11315831 10.2337/diacare.24.4.683

[8] Lakka HM, Laaksonen DE, Lakka TA, Niskanen LK, Kumpusalo E, et al (2002) The metabolic syndrome and total and cardiovascular disease mortality in middle-aged men. Jama 288: 2709–2716. 12460094 10.1001/jama.288.21.2709

[9] Ribisl PM, Lang W, Jaramillo SA, Jakicic JM, Stewart KJ, et al (2007) Exercise capacity and cardiovascular/metabolic characteristics of overweight and obese individuals with type 2 diabetes: the Look AHEAD clinical trial. Diabetes Care 30: 2679–2684. 17644623 10.2337/dc06-2487

[10] Ford ES, Li C (2006) Physical activity or fitness and the metabolic syndrome. Expert Rev Cardiovasc Ther 4: 897–915. 17173504 10.1586/14779072.4.6.897

[11] Kinjo M, Setoguchi S, Solomon DH (2007) Bone mineral density in adults with the metabolic syndrome: analysis in a population-based U.S. sample. J Clin Endocrinol Metab 92: 4161–4164. 17785365 10.1210/jc.2007-0757

[12] Wells G (2013) Metabolic syndrome and diabetes mellitus in COPD: European Respiratory Monograph.

[13] Marquis K, Maltais F, Duguay V, Bezeau AM, LeBlanc P, et al (2005) The metabolic syndrome in patients with chronic obstructive pulmonary disease. J Cardiopulm Rehabil 25: 226–232; discussion 233–224. 16056071 10.1097/00008483-200507000-00010

[14] Watz H, Waschki B, Kirsten A, Muller KC, Kretschmar G, et al (2009) The metabolic syndrome in patients with chronic bronchitis and COPD: frequency and associated consequences for systemic inflammation and physical inactivity. Chest 136: 1039–1046. 19542257 10.1378/chest.09-0393

[15] Vestbo J, Hurd SS, Agusti AG, Jones PW, Vogelmeier C, et al (2013) Global Strategy for the Diagnosis, Management, and Prevention of Chronic Obstructive Pulmonary Disease: GOLD Executive Summary. Am J Respir Crit Care Med 187: 347–365. 22878278 10.1164/rccm.201204-0596PP

[16] Jones PW, Quirk FH, Baveystock CM, Littlejohns P (1992) A self-complete measure of health status for chronic airflow limitation. The St. George's Respiratory Questionnaire. Am Rev Respir Dis 145: 1321–1327. 1595997 10.1164/ajrccm/145.6.1321

[17] Zigmond AS, Snaith RP (1983) The hospital anxiety and depression scale. Acta Psychiatr Scand 67: 361–370. 6880820 10.1111/j.1600-0447.1983.tb09716.x

[18] Charlson ME, Pompei P, Ales KL, MacKenzie CR (1987) A new method of classifying prognostic comorbidity in longitudinal studies: development and validation. J Chronic Dis 40: 373–383. 3558716 10.1016/0021-9681(87)90171-8

[19] van den Borst B, Gosker HR, Koster A, Yu B, Kritchevsky SB, et al (2012) The influence of abdominal visceral fat on inflammatory pathways and mortality risk in obstructive lung disease. Am J Clin Nutr 96: 516–526. 22811442 10.3945/ajcn.112.040774 PMC3417214

[20] Leone N, Courbon D, Thomas F, Bean K, Jego B, et al (2009) Lung function impairment and metabolic syndrome: the critical role of abdominal obesity. Am J Respir Crit Care Med 179: 509–516. 19136371 10.1164/rccm.200807-1195OC

[21] Schols AM, Mostert R, Soeters PB, Wouters EF (1991) Body composition and exercise performance in patients with chronic obstructive pulmonary disease. Thorax 46: 695–699. 1750015 10.1136/thx.46.10.695 PMC463385

[22] Wong CY, O'Moore-Sullivan T, Fang ZY, Haluska B, Leano R, et al (2005) Myocardial and vascular dysfunction and exercise capacity in the metabolic syndrome. Am J Cardiol 96: 1686–1691. 16360358 10.1016/j.amjcard.2005.07.091

[23] Yamaguchi T, Kanazawa I, Yamamoto M, Kurioka S, Yamauchi M, et al (2009) Associations between components of the metabolic syndrome versus bone mineral density and vertebral fractures in patients with type 2 diabetes. Bone 45: 174–179. 19446053 10.1016/j.bone.2009.05.003

[24] Reid IR (2006) Obesity and osteoporosis. Ann Endocrinol (Paris) 67: 125–129. 16639362 10.1016/s0003-4266(06)72567-7

[25] Reid IR (2010) Fat and bone. Arch Biochem Biophys 503: 20–27. 20599663 10.1016/j.abb.2010.06.027

[26] Pobeha P, Ukropec J, Skyba P, Ukropcova B, Joppa P, et al (2011) Relationship between osteoporosis and adipose tissue leptin and osteoprotegerin in patients with chronic obstructive pulmonary disease. Bone 48: 1008–1014. 21376149 10.1016/j.bone.2011.02.017

[27] Ferrer M, Alonso J, Morera J, Marrades RM, Khalaf A, et al (1997) Chronic obstructive pulmonary disease stage and health-related quality of life. The Quality of Life of Chronic Obstructive Pulmonary Disease Study Group. Ann Intern Med 127: 1072–1079. 9412309 10.7326/0003-4819-127-12-199712150-00003

[28] Ketelaars CA, Schlosser MA, Mostert R, Huyer Abu-Saad H, Halfens RJ, et al (1996) Determinants of health-related quality of life in patients with chronic obstructive pulmonary disease. Thorax 51: 39–43. 8658367 10.1136/thx.51.1.39 PMC472797

[29] Hajiro T, Nishimura K, Tsukino M, Ikeda A, Koyama H, et al (1998) Comparison of discriminative properties among disease-specific questionnaires for measuring health-related quality of life in patients with chronic obstructive pulmonary disease. Am J Respir Crit Care Med 157: 785–790. 9517591 10.1164/ajrccm.157.3.9703055

[30] Shoup R, Dalsky G, Warner S, Davies M, Connors M, et al (1997) Body composition and health-related quality of life in patients with obstructive airways disease. Eur Respir J 10: 1576–1580. 9230250 10.1183/09031936.97.10071576

[31] Roriz-Cruz M, Rosset I, Wada T, Sakagami T, Ishine M, et al (2007) Stroke-independent association between metabolic syndrome and functional dependence, depression, and low quality of life in elderly community-dwelling Brazilian people. J Am Geriatr Soc 55: 374–382. 17341239 10.1111/j.1532-5415.2007.01068.x

[32] Tsai AG, Wadden TA, Sarwer DB, Berkowitz RI, Womble LG, et al (2008) Metabolic syndrome and health-related quality of life in obese individuals seeking weight reduction. Obesity (Silver Spring) 16: 59–63. 18223613 10.1038/oby.2007.8

[33] Minas M, Kostikas K, Papaioannou AI, Mystridou P, Karetsi E, et al (2011) The association of metabolic syndrome with adipose tissue hormones and insulin resistance in patients with COPD without co-morbidities. COPD 8: 414–420. 22149401 10.3109/15412555.2011.619600

